# Assessment of genetic diversity using microsatellite markers to compare donkeys (*Equus asinus*) with horses (*Equus caballus*)

**DOI:** 10.5713/ab.20.0860

**Published:** 2021-04-23

**Authors:** Su Min Kim, Sung Wook Yun, Gil Jae Cho

**Affiliations:** 1 College of Veterinary Medicine, Kyungpook National University, Daegu 41566, Korea; 2 College of Veterinary Medicine and Institute of Equine Medicine, Kyungpook National University, Daegu 41566, Korea

**Keywords:** Donkey, Horse, Microsatellite Marker, South Korea

## Abstract

**Objective:**

The study aimed to evaluate the diversity of donkey populations by comparing with the diversity of Thoroughbred and Jeju Halla horses; identified breeding backgrounds can contribute to management and conservation of donkeys in South Korea.

**Methods:**

A total of 100 horse (50 Thoroughbreds and 50 Jeju Halla horses) and 79 donkeys samples were genotyped with 15 microsatellite markers (AHT4, AHT5, ASB2, ASB17, ASB23, CA425, HMS1, HMS2, HMS3, HMS6, HMS7, HTG4, HTG10, LEX3, and VHL20), to identify genetic diversity and relationships among horses and donkeys.

**Results:**

The observed number of alleles per locus ranged from 1 (ASB17, HMS1) to 14 (AHT5), with a mean value of 4.87, 8.00, and 5.87 in Thoroughbreds, Jeju Halla horses, and donkeys, respectively. Of the 15 markers, AHT4, AHT5, ASB23, CA425, HMS2, HMS3, HTG4, HTG10, and LEX3 loci had relatively high polymorphism information content (PIC) values (PIC>0.5) in these three populations. Mean levels of genetic variation were H_E_ = 0.6721 and H_O_ = 0.6600 in Thoroughbreds, H_E_ = 0.7898 and H_O_ = 0.7100 in Jeju Halla horses, and H_E_ = 0.5635 and H_O_ = 0.4861 in donkeys. Of the 15 loci in donkeys, three loci had negative inbreeding coefficients (FIS), with a moderate mean FIS (0.138). The FIS estimate for the HTG4 marker was highest (0.531) and HMS6 marker was lowest (−0.001). The total probability of exclusion value of 15 microsatellite loci was 0.9996 in donkeys.

**Conclusion:**

Genetic cluster analysis showed that the genetic relationship among 79 donkeys was generally consistent with pedigree records. Among the three breeds, donkeys and Thoroughbred horses formed clearly different groups, but the group of Jeju Halla horses overlapped with that of Thoroughbred horses, suggesting that the loci would be suitable for donkey parentage testing. Therefore, the results of this study are a valid tool for genetic study and conservation of donkeys.

## INTRODUCTION

The Equidae family includes a single genus *Equus*, which contains four subgenera with eight species. The subgenus *Equus* includes *Equus* (*E*.) *caballus* (domestic horses and Przewalski’s or Mongolian wild horses), subgenus *Asinus* includes *E. asinus* (donkeys), *E. hemionus* (Onagers and Asian wild assess), and *E. kiang* (Kiangs). The subgenus *Dolichohippus* includes only *E. grevyi* (Grevy’s zebras) and the subgenus *Hippotigris* includes *E. zebra* (mountain zebras), *E. burchelli* (Burchell’s or plains zebras), and *E. quagga* (Quaggas) [1]. The domestication of *E. asinus* is assumed to have occurred ~6,000 BC in North Africa (particularly near present-day Egypt) from Nubian and Somali wild assess [2]. For centuries, *E. asinus* has been used by humans primarily for transportation and it remains an important work animal in economically challenged areas [3]. Approximately 5.9 million *E. asinus* exist globally and most of them are economically essential in third-world countries. Unlike other whole-hoofed animals, *E. asinus* has not been sufficiently studied; they are domestically bred mostly for experience, to tow tourist carriages, or for meat; however, there is little data for bloodlines of domestically bred donkeys. At present, in South Korea, ~1,000 donkeys and ~27,000 domestic horses exist, including 12,000 Thoroughbreds, 1,000 individuals from other horse breeds (e.g., Warmblood, Quarter horse), and 14,000 native horses (the Jeju Halla horse), of which ~5,000 Jeju horses which was designated as natural monument No. 347 by the government [4].

DNA analysis using microsatellite markers has recently been used in many animal species for identification of individuals, paternity tests, preservation of endangered animals, and phylogenesis based on origin and breeding history. In addition, countries throughout the world have widely used microsatellite markers since the mid-1990s for the purposes of examining genetic diversity of domesticated animals, tracking their origin and inherited characteristics, and promoting their preservation [5,6].

Microsatellites are numerous repeats of simple base pair sequences in the genome of an organism, and are widely distributed in short repetitive pieces [7]. Microsatellites have a high mutation rate, over 1/10^4^ to 1/10^6^ per generation, and have high specificity to a group; polymorphisms can occur at the individual level as well as at the species level, providing a useful tool for genetic mapping as well as information about heritability of many species of plants and animals including humans [8–12]. Microsatellites commonly have been used for the assessment of genetic diversity, construction of genetic maps, quantitative trait loci mapping, and parentage testing [13,14]. Microsatellites in a horse were reported for the first time by Ellegren et al [8] and Marklund et al [11].

The study aimed to evaluate the diversity of donkey populations by comparing with the diversity of Thoroughbred horses and Jeju Halla horses; identified breeding backgrounds can contribute to management and conservation of donkeys in South Korea.

## MATERIALS AND METHODS

### Sample collection and DNA extraction

Genomic DNA was extracted using a MagExtractor System MFX-2000 (Toyobo, Osaka, Japan) according to the manufacturer’s protocols [12] from whole blood samples of 79 donkeys and 100 horses (50 Thoroughbreds and 50 Jeju Halla horses [a hybrid cross between Thoroughbreds and Jeju horses]).

### Microsatellite markers and analysis

A total of 15 microsatellite loci (AHT4, AHT5, ASB2, ASB17, ASB23, CA425, HMS1, HMS2, HMS3, HMS6, HMS7, HTG4, HTG10, LEX3, and VHL20) were used for analysis of the Equidae. Polymerase chain reaction (PCR) was performed according to the manufacturer’s protocols (Stockmarks, Applied Biosystems, Foster, CA, USA). Of the 15 markers, ASB17, ASB23, CA425, HMS1, and LEX3 markers were conducted by a single PCR.

Multiplex PCR was accomplished using a total volume of 15 μL of the following mixture: 40 ng genomic DNA, each primer, 1.25 mM dNTPs, 2.5 μL 10× reaction buffer, and 5 U *Taq* polymerase (Applied Biosystems, USA). For a single PCR, 2 μL template DNA, 2 μL of both 10 pmol forward and reverse primers, and 6.5 μL sterile distilled water were mixed in PCR Premix buffer (Qiagen, Hidden, Germany), adjusted to 25 μL in total.

PCR amplification was as follows: initial denaturation for 10 min at 95°C, followed by 30 cycles at 95°C for 30 s, 60°C for 30 s, and 72°C for 1 min. An extension step at 72°C for 60 min was added after the final cycle [15]. Multiplex PCR reactions were performed in a GeneAmp PCR System 9700 (Applied Biosystems, USA).

PCR products were tested using an automatic gene analyzer (ABI 3130 xl Genetic Analyzer, Foster, CA, USA); subsequent electrophoresis was done on POP 7 polymer (Applied Biosystems, USA) at 15 kV. Using peak row data, the size of alleles (in base pairs) for each marker was determined based on the results of 2015/2016 Horse Comparison Test No. 1 of the International Society for Animal Genetics (ISAG), using GeneMapper Software ver. 4.0 (Applied Biosystems, USA).

### Statistical analysis

Allelic frequencies and the number of alleles per locus were estimated by direct counting from the observed genotype; the observed heterozygosity (H_O_), expected heterozygosity (H_E_), number of allelic genes and frequency, and polymorphism information content (PIC) value for each breed across the locus were calculated using Cervus ver. 3.0.3 [16].

The inbreeding coefficient of an individual relative to the subpopulation (FIS) was calculated using FSTAT (Ver. 2.9.3; Goudet, 2001). In addition, we analyzed the genetic distance of each individual based on the shared allele distance using the Microsat package.

## RESULTS

### Analysis of genetic diversity

In our study, 15 microsatellites were used to identify genetic diversity and relationships among horses and donkeys. The observed number of alleles per locus ranged from 1 (ASB17, HMS1) to 14 (AHT5), with a mean value of 4.87, 8.00, and 5.87 in Thoroughbreds, Jeju Halla horses, and donkeys, respectively. Of the 15 markers, AHT4, AHT5, ASB23, CA425, HMS2, HMS3, HTG4, HTG10, and LEX3 loci had relatively high PIC values (PIC>0.5) in these three species. Mean levels of genetic variation were as follows: Thoroughbred, H_O_ = 0.6600 and H_E_ = 0.6721; Jeju Halla, H_O_ = 0.7100 and H_E_ = 0.7898; and donkey, H_O_ = 0.4861 and H_E_ = 0.5635 (Table 1). Of the 15 loci in the donkey population, three loci had negative inbreeding coefficients and the mean FIS was moderate (0.138). The FIS estimate for HTG4 marker was highest (0.531) and HMS6 marker was lowest (−0.001). The total probability of exclusion value of 15 microsatellite loci was 0.9996 in the donkey population (Table 2).

### Population relationship

**Figure 1:**
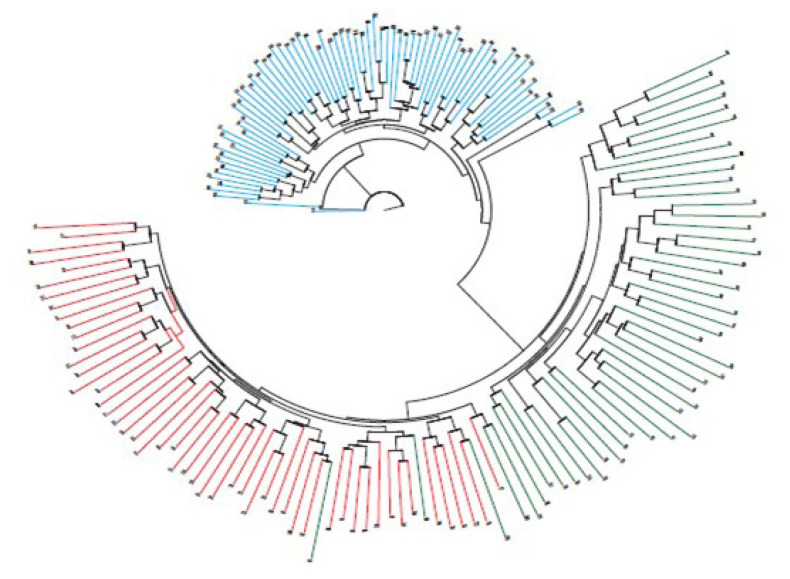
A neighbor-joining dendrogram, constructed from allele-sharing distances among 179 individuals in donkeys and two horse breeds. Blue color, donkey; Green color, Thoroughbred; Red color, Jeju Halla horse.

Using simple allele-sharing levels to calculate the genetic differences between donkey and horse breeds, a collective genetic analysis was based on individual allele frequency using the analysis with Microsat (Figure 1). Genetic cluster analysis showed that the genetic relationship among 79 donkeys was generally consistent with pedigree records. Among the three breeds, donkeys and Thoroughbreds formed clearly different groups; the Jeju Halla horse formed a group that overlapped with Thoroughbred horses.

## DISCUSSION

Many microsatellite markers have previously been isolated from the horse genome; these microsatellites showed multiple alleles as well as high heterozygosity among European horse breeds such as the Thoroughbred horse [17]. Microsatellites are informative due to their high rates of polymorphism and are useful in paternity testing of animals [13,18–22]; they have been used extensively to examine the structure of closely related populations and breed allocation of animals [13,20,22–24]. In cattle, pigs, horses, and dogs, pedigree control has been performed on a routine basis in most countries. These controls rely on microsatellite typing that has been standardized through regular comparison tests under the auspices of ISAG.

In South Korea, to meet the demands of the domestic donkey market and secure a better quality of donkeys and donkey meats, it is necessary to select donkeys with excellent pedigrees to begin and enhance donkey breeding. However, currently, in South Korea, investigations of the breeding and genetics of donkeys, compared with other breeds such as the Thoroughbred, the Jeju horse, or the Jeju Halla horse, are insufficient.

Because several loci in the current study had relatively high PIC values (PIC>0.5) in populations examined, it is likely that these markers can be utilized in the differentiation of donkey individuals and for paternity tests. Based on the PIC value of each marker, the validity and reliability of the marker can be estimated; if the PIC value is >0.5000, the reliability of the marker is valid for pedigree analysis. If the PIC value is >0.7000, it has universal validity for analysis and has a high reliability. An international panel of 13 markers (AHT4, ASB23, HMS2, HMS3, HMS6, HMS7, HMS18, HTG7, HTG10, TKY297, TKY312, TKY337, and TKY343) for donkeys has recently been recommended by ISAG. However, donkeys are raised primarily in the country in which they exist, unlike Thoroughbred horses that are extensively imported and exported; therefore, the nine microsatellite markers identified in this study are considered appropriate for individual identification and parentage verification.

In a single gene locus, an indicator of diversity is heterozygosity. In an association analysis or linkage disequilibrium analysis, higher heterozygosity is more desirable [9,25,26]. When multiple groups are mixed, heterozygosity increases, but if there is no interbreeding of groups, heterozygosity is generally related to mutations in the population [27]. For analysis of genetic characteristics using a microsatellite marker, heterozygosity can be judged from the degree of mixing between target populations and other populations. If a pure pedigree is preserved through powerful selection without a mix of species, the value of heterozygosity is low; if there is a mix of different breeds, heterozygosity is high. However, when more individuals are used in a study, heterozygosity is higher, so it may be difficult to judge the mix of species based only on heterozygosity. A collective genetic analysis based on individual allele frequencies indicates that donkeys and Thoroughbred horses formed clearly different groups, but Jeju Halla horses formed a group that overlapped with that of Thoroughbred horses. Jeju Halla horses are a hybrid of Thoroughbred horses and Jeju horses; the inclusion of five Thoroughbred horses within the pedigree likely caused an appearance of their genetic factors in the present analysis.

The donkey had the lowest heterozygosity compared with horse breeds; the FIS of 0.138 indicates that genetic diversity is decreasing due to inbreeding. Low heterozygosity is likely due to few breeding herds of domesticated donkeys and inbreeding produced by a limited number of male horses. Therefore, a breeding program is needed to minimize inbreeding within farms and more stallions should be used for genetic diversity of domestically bred donkeys.

Donkeys in South Korea are less abundant compared with horse breeds, but they are very valuable in riding tourism and meat. Therefore, to secure and to preserve genetic diversity of donkeys, systematic management through the selection of stallions and registration of pedigrees are necessary. The evaluation of genetic diversity of donkeys suggests that nine microsatellite markers (AHT4, AHT5, ASB23, CA425, HMS2, HMS3, HTG4, HTG10, and LEX3) could be used for individual identification and parentage verification in donkeys. Although it is currently under discussion whether to use single nucleotide polymorphisms instead of microsatellite DNA markers for parentage verification and individual identification of horses, the results of this study suggest that microsatellites are also valid as a tool for the genetic study and conservation of donkeys.

## Figures and Tables

**Table 1:** Number of alleles, heterozygosity, and polymorphism information content of 15 microsatellite markers in 179 donkeys and horses

Marker	No. of alleles			OHet			EHet			PIC		
			
	DK	TB	JH	DK	TB	JH	DK	TB	JH	DK	TB	JH
AHT4	6	4	6	0.6076	0.7800	0.6400	0.7102	0.6919	0.8081	0.6547	0.6547	0.7686
AHT5	13	5	7	0.8228	0.6800	0.7600	0.9104	0.6440	0.8008	0.8968	0.5882	0.7634
ASB2	2	6	8	0.4557	0.7600	0.9200	0.4605	0.8135	0.8121	0.3529	0.7777	0.7755
ASB17	1	5	13	0.0000	0.6400	0.7400	0.0000	0.7206	0.8749	0.0000	0.6675	0.6675
ASB23	6	6	8	0.8608	0.8000	0.7600	0.7364	0.7966	0.8208	0.6969	0.7562	0.7562
CA425	8	5	9	0.7722	0.5200	0.7400	0.7364	0.5057	0.7970	0.6839	0.4544	0.7577
HMS1	1	3	8	0.0000	0.5200	0.7600	0.0000	0.6317	0.6473	0.0000	0.5465	0.5742
HMS2	8	5	9	0.7215	0.3400	0.6200	0.7447	0.3903	0.7570	0.6989	0.3617	0.7076
HMS3	6	5	7	0.6456	0.5800	0.5400	0.6177	0.6519	0.7887	0.5473	0.5960	0.7501
HMS6	3	4	7	0.5063	0.6400	0.6400	0.5059	0.6236	0.7644	0.4043	0.5655	0.7167
HMS7	4	5	6	0.2025	0.8400	0.6800	0.2320	0.7697	0.7661	0.2203	0.7214	0.7189
HTG4	8	4	5	0.3544	0.5800	0.6600	0.7819	0.5562	0.6844	0.7461	0.4610	0.6276
HTG10	8	6	9	0.6456	0.8600	0.8000	0.7368	0.8158	0.8016	0.7000	0.7811	0.7648
LEX3	11	6	9	0.3671	0.6200	0.6600	0.7762	0.7321	0.8570	0.7439	0.6800	0.8315
VHL20	3	4	9	0.3291	0.7200	0.7200	0.5041	0.7380	0.8673	0.3875	0.6811	0.8427
Mean	5.87	4.87	8.00	0.4861	0.6600	0.7100	0.5635	0.6721	0.7898	0.5156	0.6195	0.7349

OHet, observed heterozygosity; EHet, expected heterozygosity; PIC, polymorphism information content; DK, donkey; TB, thoroughbred; JH, Jeju Halla horse (crossbred).

**Table 2:** Statistical analysis of gene diversity per locus, inbreeding coefficient, and exclusion probability in 79 donkeys

Locus	Gene diversity	FIS	PE (1)	PE (2)
AHT4	0.711	0.145	0.288	0.458
AHT5	0.878	0.106	0.666	0.800
ASB2	0.461	0.011	0.105	0.176
ASB17	0.000	0.000	0.000	0.000
ASB23	0.740	−0.163	0.336	0.514
CA425	0.736	−0.049	0.317	0.489
HMS1	0.000	0.000	0.000	0.000
HMS2	0.745	0.031	0.337	0.513
HMS3	0.617	−0.045	0.205	0.352
HMS6	0.506	−0.001	0.126	0.218
HMS7	0.232	0.128	0.027	0.122
HTG4	0.782	0.531	0.400	0.578
HTG10	0.723	0.124	0.325	0.527
LEX3	0.779	0.529	0.400	0.581
VHL20	0.505	0.349	0.125	0.201
Total	0.561*	0.138	0.991	0.999

FIS, inbreeding coefficient; PE (1), total exclusionary power (first parent); PE (2), total exclusionary power (second parent).
